# Incidence of pneumonitis with CTLA-4 inhibitors in non-small cell lung cancer: a systematic review and meta-analysis

**DOI:** 10.3389/fmed.2025.1614442

**Published:** 2025-08-06

**Authors:** Wei Li, Hao Xiong, Haiying Peng, Ting Yang, Li Fan, Yanlin Zhang

**Affiliations:** ^1^Department of Pathology, Second People’s Hospital of Yibin, Yibin, Sichuan, China; ^2^Department of Respiratory and Critical Care Medicine, Second People’s Hospital of Yibin, Yibin, Sichuan, China

**Keywords:** CTLA-4 inhibitors, non-small cell lung cancer (NSCLC), meta-analysis, pneumonitis, immune-related adverse events, tremelimumab, ipilimumab, immune checkpoint inhibitors

## Abstract

**Background:**

CTLA-4 inhibitors, such as tremelimumab and ipilimumab, are increasingly used in the treatment of non-small cell lung cancer (NSCLC). This meta-analysis aims to evaluate the incidence of pneumonitis associated with these inhibitors and explore potential differences between individual agents.

**Methods:**

A systematic search across three online databases identified 911 records. After screening for duplicates and irrelevant articles, nine studies with a total of 4,164 patients were included. Risk of bias was assessed using the Cochrane “Risk of Bias” tool. Pneumonitis incidence was analyzed using a random-effects model.

**Results:**

The overall incidence of any-grade pneumonitis was 4.0% [95% CI (2.2%, 5.8%)]. High-grade pneumonitis occurred in 1.6% [95% CI (0.5%, 2.6%)]. Subgroup analysis revealed that tremelimumab was associated with a higher incidence of both any-grade (8.0% vs. 2.0%) and high-grade (3.0% vs. 1.0%) pneumonitis compared to ipilimumab. In a comparison with a control group, patients receiving CTLA-4 inhibitors had a significantly higher incidence of any-grade pneumonitis [OR = 3.00, 95% CI (1.60, 5.64), *p* < 0.01]. However, the difference in high-grade pneumonitis between the two groups was not statistically significant [RR = 1.79, 95% CI (0.83, 3.85), *p* = 0.14].

**Conclusion:**

This meta-analysis indicates that CTLA-4 inhibitors are associated with a higher incidence of pneumonitis in NSCLC patients, particularly with tremelimumab. These findings underline the importance of close monitoring for pneumonitis in patients receiving CTLA-4 inhibitors, especially tremelimumab, and suggest the need for further research into prevention and management strategies.

## 1 Introduction

Immune evasion is increasingly recognized as a key hallmark of lung cancer progression (1). The activation of immune checkpoint pathways, such as Programmed Death-1 (PD-1)/Programmed Death- Ligand 1 (PD-L1) and Cytotoxic T-Lymphocyte Antigen 4 (CTLA-4), represents a crucial mechanism by which tumor cells evade immune surveillance (2). Immune checkpoint inhibitors (ICIs) block the interaction between these checkpoint proteins, thereby disrupting the immune balance in favor of enhancing immune responses against tumors (3, 4).

Clinical studies have demonstrated significant advancements in Progression-Free Survival (PFS) and Overall Survival (OS) with the application of ICIs, particularly in patients with advanced non-small cell lung cancer (NSCLC) (5–7). To date, several ICIs have been approved by regulatory agencies for the treatment of advanced NSCLC, including PD-1 inhibitors (nivolumab, pembrolizumab), PD-L1 inhibitors (atezolizumab, durvalumab), and the CTLA-4 inhibitor ipilimumab (8).

The CTLA-4 and PD-1 pathways play distinct roles at different stages of the immune response. CTLA-4 acts during the initial phase of T-cell activation, typically in the lymph nodes, to inhibit the activation of potentially self-reactive T-cells. In contrast, the PD-1 pathway primarily modulates previously activated T-cells in peripheral tissues during later stages of the immune response (9). Consequently, simultaneous blockade of both the CTLA-4 and PD-1 pathways results in higher efficacy than blocking either pathway alone or sequentially (10, 11).

However, this enhanced immune activation also leads to increased immune-related adverse events (irAEs), including checkpoint inhibitor pneumonitis (CIP), a rare but potentially fatal toxicity (12–14). While PD-1/PD-L1 inhibitors are more commonly used as first-line therapies in NSCLC, CTLA-4 inhibitors are often used in combination regimens. Notably, dual blockade involving CTLA-4 is associated with a higher incidence of irAEs, including CIP (15). CIP occurs more frequently and rapidly in NSCLC compared to other cancers (16), making its clinical management especially challenging. Although several studies have examined CIP in the context of PD-1/PD-L1 inhibitors, the specific contribution of CTLA-4 blockade to CIP risk, especially when used in monotherapy or combination regimens remains unclear. This systematic review and meta- analysis aims to evaluate the incidence of CIP specifically associated with CTLA-4 inhibitors in NSCLC, and to explore differences across individual agents. Future comparative analysis with PD-1/PD-L1-related CIP could further contextualize these findings and inform clinical decision-making.

## 2 Methods

### 2.1 Literature search

This study adheres to the Preferred Reporting Items for Systematic Reviews and Meta-Analyses (PRISMA) guidelines (17). Two researchers independently conducted a systematic literature search in PubMed, Embase, and the Cochrane Central Register of Controlled Trials to identify eligible randomized controlled trials published from 2015 to 2024 (up to 26 July 2024). The included studies were required to involve at least one group receiving a CTLA-4 inhibitor approved by the United States Food and Drug Administration (FDA) for the treatment of NSCLC. Only studies published in English were considered.

### 2.2 Inclusion and exclusion criteria

#### 2.2.1 Inclusion criteria

1. Patients with pathologically diagnosed NSCLC at stages I, II, III, or IV.
2. Treatment with a CTLA-4 inhibitor either as monotherapy or in combination.
3. Reporting of the incidence of grades 1–5 and grades 3–5 CIP.

#### 2.2.2 Exclusion criteria

1. Non-randomized controlled trials.
2. Absence of relevant study data.

### 2.3 Data extraction and risk of bias assessment

Data extracted from the studies included: first author, publication year, country of publication, patient age, treatment regimen, prior treatment history, the number of patients treated with CTLA-4 inhibitors, type of immunotherapy used, the number of patients experiencing immunotherapy-related pneumonitis at grades 1–5 and 3–5, and follow-up duration (Supplementary Table 1).

The quality of the included studies was assessed using the “Risk of Bias” tool from the Cochrane Review Manager, as per the Cochrane Handbook (17). Discrepancies between the two researchers were resolved through discussion with a third researcher.

### 2.4 Outcome measures

The primary outcome of this study was the incidence of grade 1–5 CIP (any-grade), and the secondary outcome was the incidence of grade 3–5 (high-grade) immune-related pneumonitis.

### 2.5 Statistical analysis

A meta-analysis was conducted using the Review Manager (RevMan) online platform using RevMan 5.4.1 version and STATA 12.0 software. Heterogeneity was assessed using Cochran’s Q test and the *I*^2^ statistic. Studies with low heterogeneity (*I*^2^ < 50%, *p* > 0.05) were analyzed using a fixed-effect model, while studies with high heterogeneity (*I*^2^ ≥ 50%, *p* < 0.05) were analyzed using a random-effects model. Effect sizes were expressed as event rates with 95% confidence intervals (CIs). *P*-value of 0.05 or less considered as significant and limit of less than 0.01 as limit.high Publication bias was assessed using funnel plots. Forest plots illustrating the prevalence of CIP were generated, and subgroup analyses were conducted to explore potential differences between individual CTLA-4 inhibitors. To explore sources of heterogeneity, a leave-one-out sensitivity analysis was conducted by sequentially excluding each study to assess its impact on the overall I^2^ statistic and pooled effect size. If heterogeneity remained high, subgroup analyses were performed to investigate potential sources, such as differences in CTLA-4 inhibitor type (tremelimumab vs. ipilimumab).

## 3 Results

### 3.1 Study selection and characteristics

**FIGURE 1:**
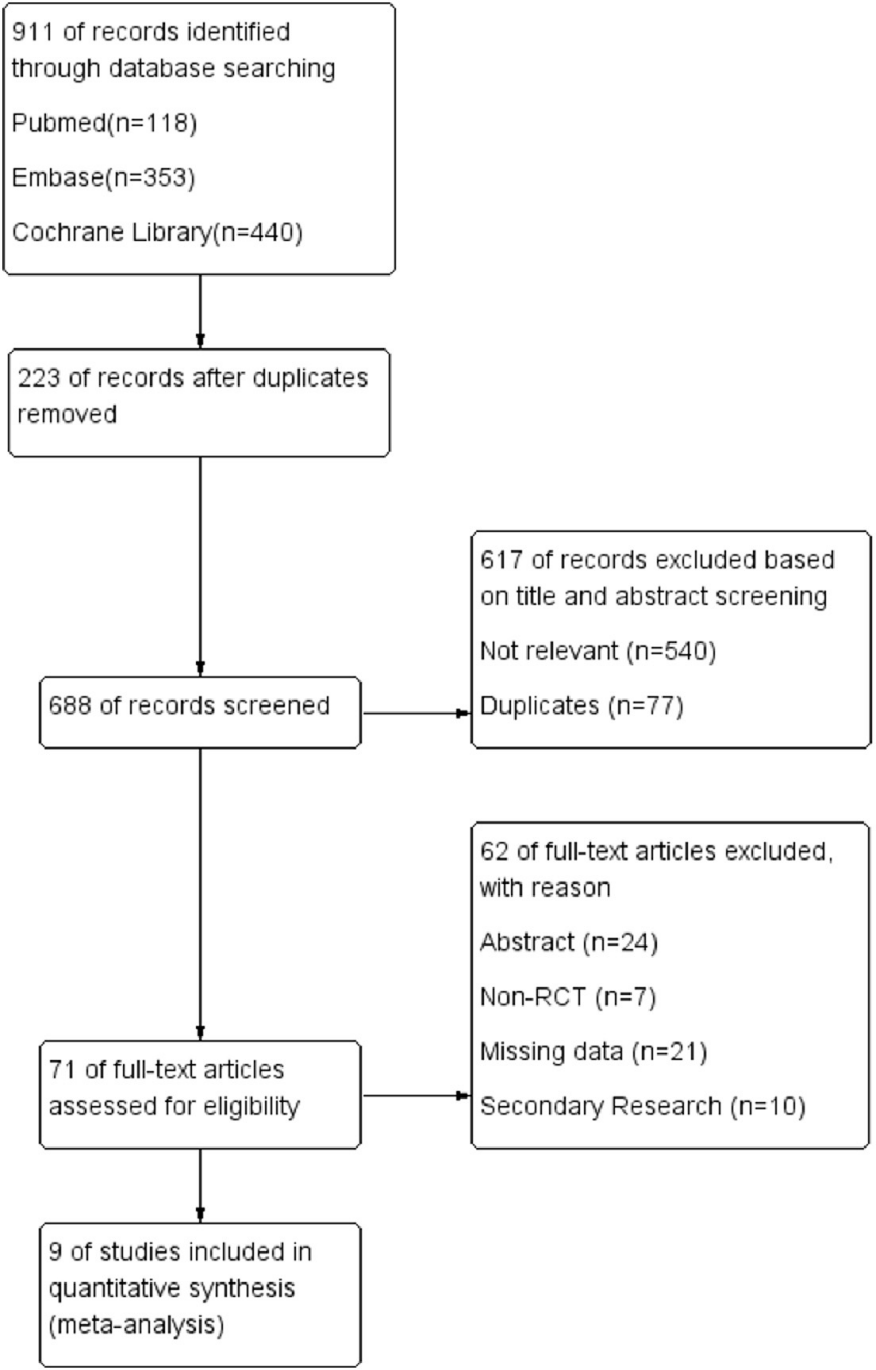
Preferred Reporting Items for Systematic Reviews and Meta-Analyses (PRISMA) flow diagram showing the study selection process. From 911 records identified through database searching, nine studies with 4,164 patients were finally included after removing duplicates and applying inclusion/exclusion criteria.

A preliminary search across three online databases yielded a total of 911 records. After identifying and excluding 223 duplicate articles, further screening based on titles and abstracts led to the removal of an additional 77 duplicates and 540 irrelevant articles. The remaining 71 articles were fully retrieved and assessed for eligibility, resulting in the exclusion of 62 articles. The reasons for exclusion included secondary research such as systematic reviews, meta-analyses, and narrative reviews (*n* = 10). The PRISMA study selection flowchart is shown in Figure 1.

The baseline data and relevant characteristics of the studies meeting the inclusion criteria are summarized in Table 1, and detailed in Supplementary Table 2. This meta-analysis includes a total of 9 studies conducted between 2015 and 2024, with a cumulative sample size of 4,164 patients. The CTLA-4 inhibitors evaluated in this study include tremelimumab and ipilimumab.

**TABLE 1:** Characteristics of included studies evaluating CTLA-4 inhibitors and associated pneumonitis adverse events.

References	Study type	Phase	Agents	No. patients	Total	Pneumonitis grade 1–5	Grade 3–5
Zhao et al. (20)	RCT	Ib	IBI310 1 mg/kg + sintilimab	15	30	2	0
			IBI310 3 mg/kg + sintilimab	15	–	3	0
Schoenfeld et al. (21)	RCT	II	Durvalumab + tremelimumab + hypofractionated radiotherapy	26	78	1	1
			Durvalumab + tremelimumab + low-dose radiotherapy	26	–	0	0
			Durvalumab–tremelimumab alone	26	–	1	1
Rizvi et al. (22)	RCT	III	Durvalumab Monotherapy	163	488	8	5
			Durvalumab + Tremelimumab	163	–	25	11
			Chemotherapy	162	–	5	2
Paz-Ares et al. (19)	RCT	III	Nivolumab + ipilimumab + two cycles of chemotherapy group	361	719	1	1
			Chemotherapy group	358	–	0	0
Hellmann et al. (23)	RCT	I	Nivolumab 3 mg/kg every 2 weeks + ipilimumab 1 mg/kg every 12 weeks	38	78	4	2
			Nivolumab 3 mg/kg every 2 weeks + ipilimumab 1 mg/kg every 6 weeks	40	–	2	1
Cheng et al. (24)	RCT	III	Durvalumab + tremelimumab	78	160	11	5
			Chemotherapy	82	–	3	3
Carbone et al. (25)	RCT	III	Nivolumab + ipilimumab + chemotherapy	361	719	21	10
			Chemotherapy	358	–	–	–
Govindan et al. (18)	RCT	III	Chemotherapy + Ipilimumab	388	749	1	1
			Chemotherapy + Placebo	361	–	2	2
Leighl et al. (26)	RCT	III	Durvalumab + Tremelimumab + Chemotherapy	151	301	9	3
			Durvalumab + Tremelimumab	150	–	9	4

RCT, randomized controlled trial.

### 3.2 Risk of bias and publication bias assessment

**FIGURE 2:**
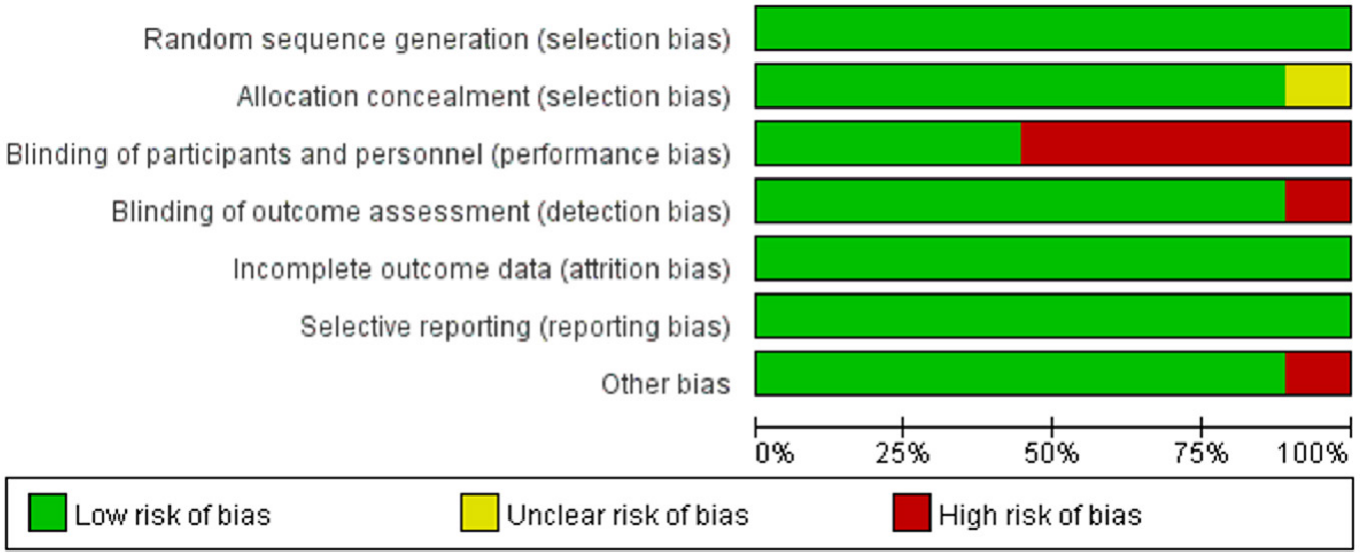
Summary of risk of bias assessment across all included studies. Most studies showed low risk of bias, with the majority of potential bias arising from the open-label design in the domain of blinding of participants and personnel.

**FIGURE 3:**
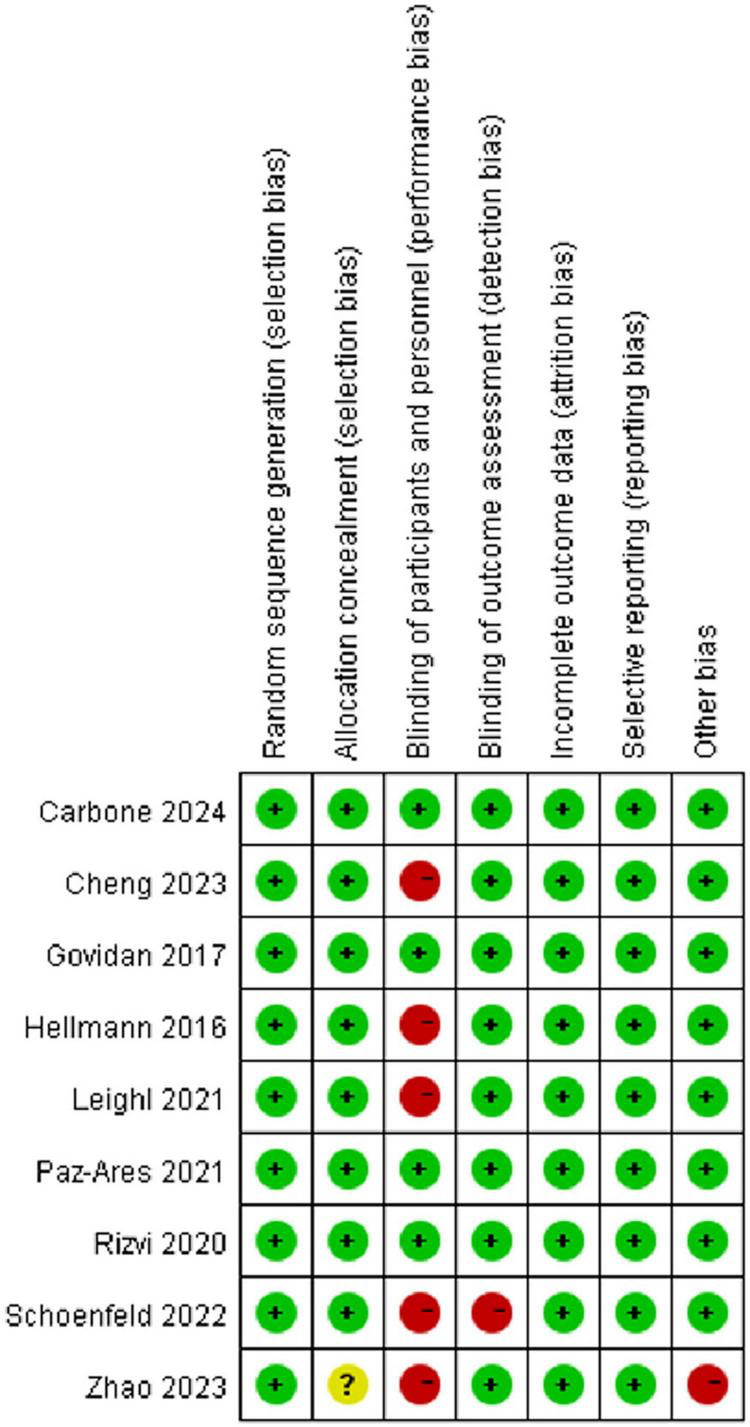
Risk of bias assessment for individual studies using the revised Cochrane Risk of Bias tool for randomized controlled trials. Seven studies demonstrated a low risk of bias, while two studies exhibited a higher risk of bias.

The Cochrane Risk of Bias tool was used to evaluate bias across included studies. Figure 2 presents the risk of bias summary for each domain, and Figure 3 shows individual study assessments. Most studies (*n* = 7) demonstrated low risk of bias overall. However, the majority were open-label trials, resulting in high risk of performance bias due to the lack of blinding of participants and personnel. Two studies exhibited a higher overall risk of bias across multiple domains. Formal assessment of publication bias using funnel plots was not feasible due to the limited number of included studies (*n* = 9), which falls below the threshold recommended for reliable asymmetry detection (17) (Supplementary Figure 2). Nevertheless, publication bias remains a concern, as studies reporting higher pneumonitis rates or statistically significant findings are more likely to be published. This potential bias may particularly affect the representativeness of findings for agents with fewer available studies, such as ipilimumab, and could limit the generalizability of our results.

### 3.3 Incidence of pneumonitis with CTLA-4 inhibitors

**FIGURE 4:**
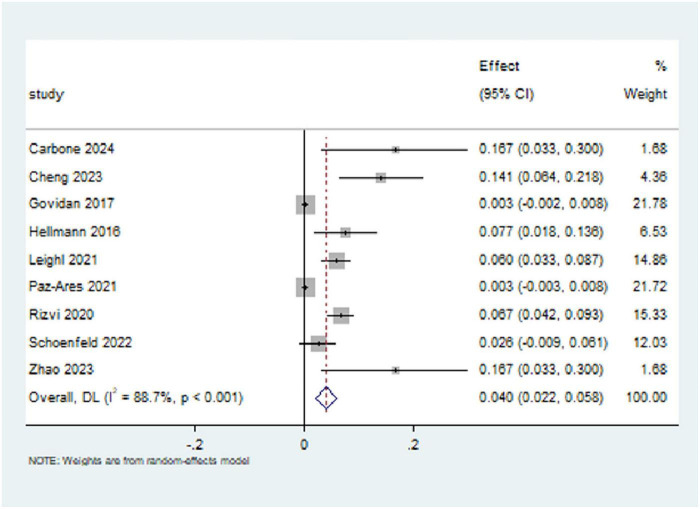
Forest plot for the incidence of any-grade pneumonitis with CTLA-4 inhibitors. The overall incidence was 4.0% (95% CI [2.2%, 5.8%]) with substantial heterogeneity (I^2^ = 88.7%).

All nine studies included in this research reported the incidence of pneumonitis (all grades) in patients with NSCLC treated with CTLA-4 inhibitors. The results indicate that the overall incidence of pneumonitis (all grades) among NSCLC patients receiving CTLA-4 inhibitors was 4.0% [95% CI (2.2%, 5.8%)]. The heterogeneity across studies was substantial, with an I^2^ value of 88.7%, indicating a high degree of variability. As a result, a random-effects model was used (*p* < 0.01), as shown in Figure 4.

#### 3.3.1 Sensitivity analysis for heterogeneity

To investigate the sources of substantial heterogeneity (I^2^ = 88.7%, *p* < 0.01) observed in the incidence of any-grade pneumonitis, a leave-one-out sensitivity analysis was conducted by sequentially excluding each included study. The results showed that the exclusion of any single study did not substantially reduce heterogeneity. However, excluding Govindan et al. (18), Paz-Ares et al. (19) reduced heterogeneity to I^2^ = 46%, suggesting that differences in study design, patient populations, or treatment regimens in these studies may have contributed to the observed variability (Supplementary Figure 1) For instance, Govindan et al. (18) evaluated ipilimumab with chemotherapy, while Paz-Ares et al. (19) (CheckMate 9LA) combined nivolumab, ipilimumab, and chemotherapy, potentially introducing variability in pneumonitis reporting or patient characteristics. However, the sensitivity analysis alone did not fully explain the heterogeneity, necessitating subgroup analysis by drug type, which identified higher pneumonitis incidence with tremelimumab compared to ipilimumab. Future studies should employ meta-regression or additional subgroup analyses to explore other potential sources of heterogeneity, such as NSCLC stage or prior treatment history.

**FIGURE 5:**
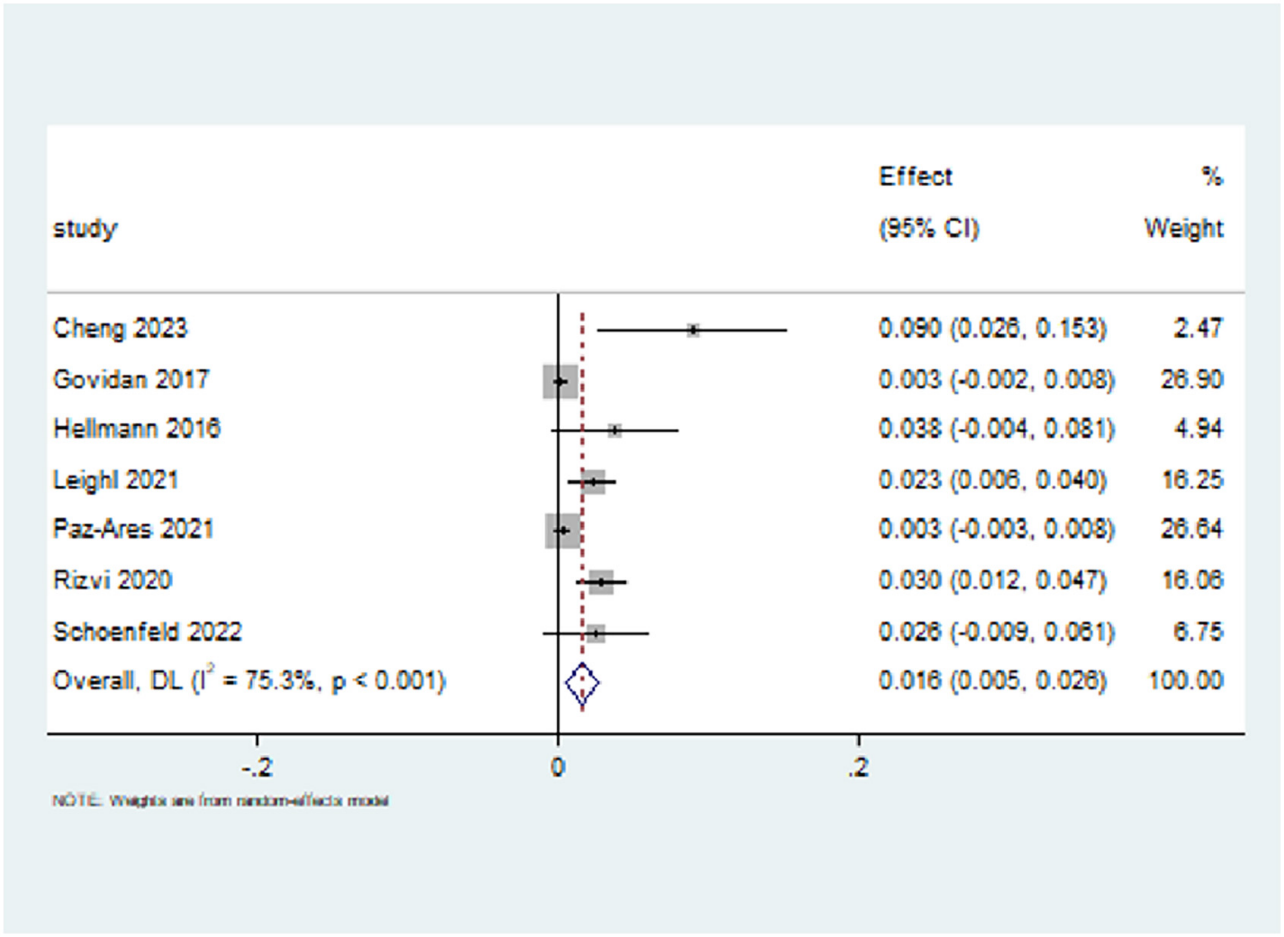
Forest plot for the incidence of high-grade (grades 3-5) pneumonitis with CTLA-4 inhibitors. The overall incidence was 1.6% (95% CI [0.5%, 2.6%]) with significant heterogeneity (I^2^ = 75.3%).

Of the studies included, seven reported the incidence of high-grade (grades 3–5) pneumonitis. The incidence of high-grade pneumonitis among NSCLC patients treated with CTLA-4 inhibitors was 1.6% ([95% CI (0.5%, 2.6%)]. The heterogeneity among the studies was significant, with an I^2^ value of 75.3%, indicating substantial variability. As a result, a random-effects model was applied (*p* < 0.001), as shown in Figure 5. Additionally, among the nine included studies, only one evaluated CTLA-4 inhibitor monotherapy, while the remaining eight involved combination regimens (e.g., CTLA-4 plus PD-1/PD-L1 inhibitors or chemotherapy). Due to this imbalance, a formal subgroup meta-analysis comparing monotherapy versus combination therapy was not feasible. Nevertheless, descriptive evaluation showed that the incidence of pneumonitis in the monotherapy arm was notably lower (approximately 1.2%), aligning with previous findings from melanoma studies (27, 28). In contrast, combination regimens were associated with higher pneumonitis rates, suggesting a potential additive or synergistic effect on pulmonary toxicity.

### 3.4 Comparison with control group

Among the included studies, four included a control group (the control arms varied across trials and included chemotherapy, placebo, or non-CTLA-4 immunotherapy) so we consider them as patients who did not receive CTLA-4 inhibitors. A comparison of pneumonitis incidence rates between patients who received CTLA-4 inhibitors and those who did not revealed that, out of 1,198 patients treated with CTLA-4 inhibitors, 38 (3.17%) experienced any-grade pneumonitis. In contrast, the control group, which consisted of 1,153 patients, had 13 (1.11%) cases of any-grade pneumonitis.

**FIGURE 6:**
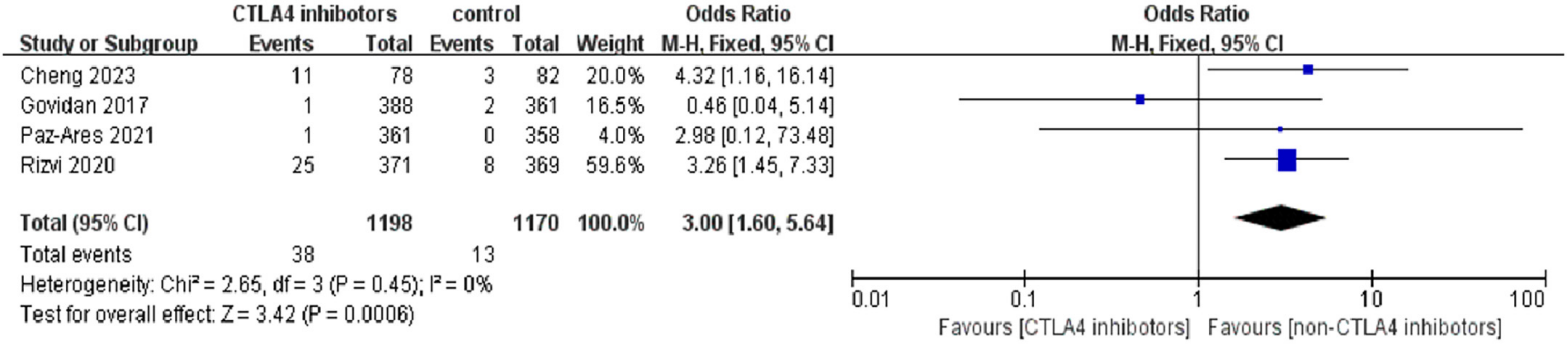
Forest plot comparing the incidence of any-grade pneumonitis between CTLA-4 inhibitor and control groups. Patients receiving CTLA-4 inhibitors had a significantly higher incidence of any-grade pneumonitis [OR = 3.00, 95% CI (1.60, 5.64), *p* < 0.01].

**FIGURE 7:**
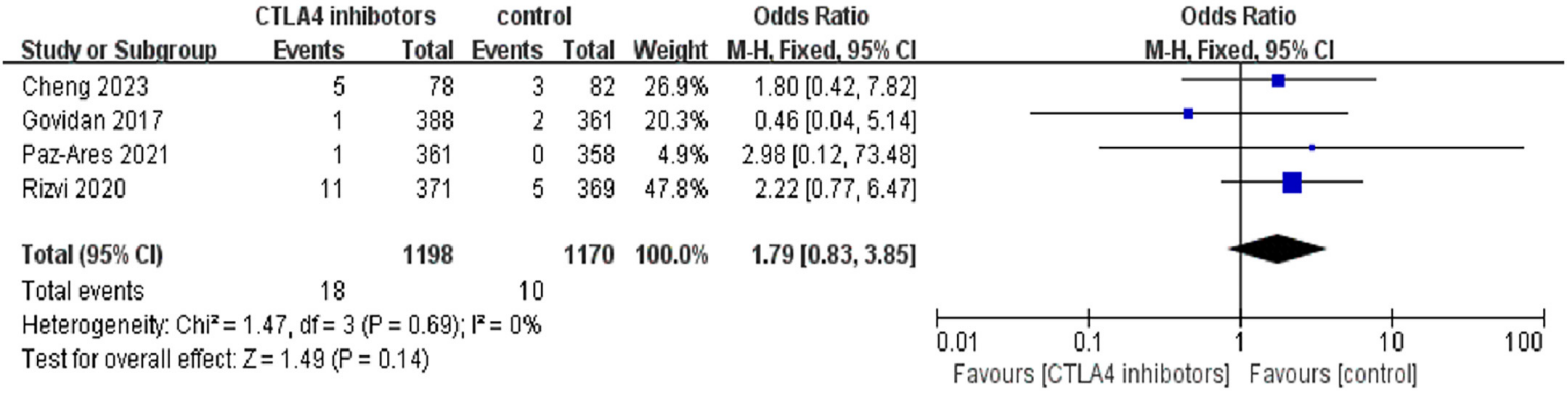
Forest plot comparing the incidence of high-grade (grade 3) pneumonitis between CTLA-4 inhibitor and control groups. While the risk ratio suggested a higher incidence in the CTLA-4 inhibitor group [RR = 1.79, 95% CI (0.83, 3.85)], the difference was not statistically significant (*p* = 0.14).

The odds ratio (OR) was 3.00 [95% CI (1.60, 5.64); *p* < 0.01], indicating a significantly higher incidence of any-grade pneumonitis in patients treated with CTLA-4 inhibitors compared to the control group (Figure 6). Additionally, 18 patients (1.50%) in the CTLA-4 inhibitor group developed high-grade pneumonitis, while 10 (0.85%) in the control group experienced high-grade pneumonitis. The risk ratio (RR) was 1.79 [95% CI (0.83, 3.85); *P* = 0.14], suggesting a higher incidence of high-grade pneumonitis in patients treated with CTLA-4 inhibitors, though the difference was not statistically significant (Figure 7).

### 3.5 Subgroup analysis by CTLA-4 inhibitor type

**FIGURE 8:**
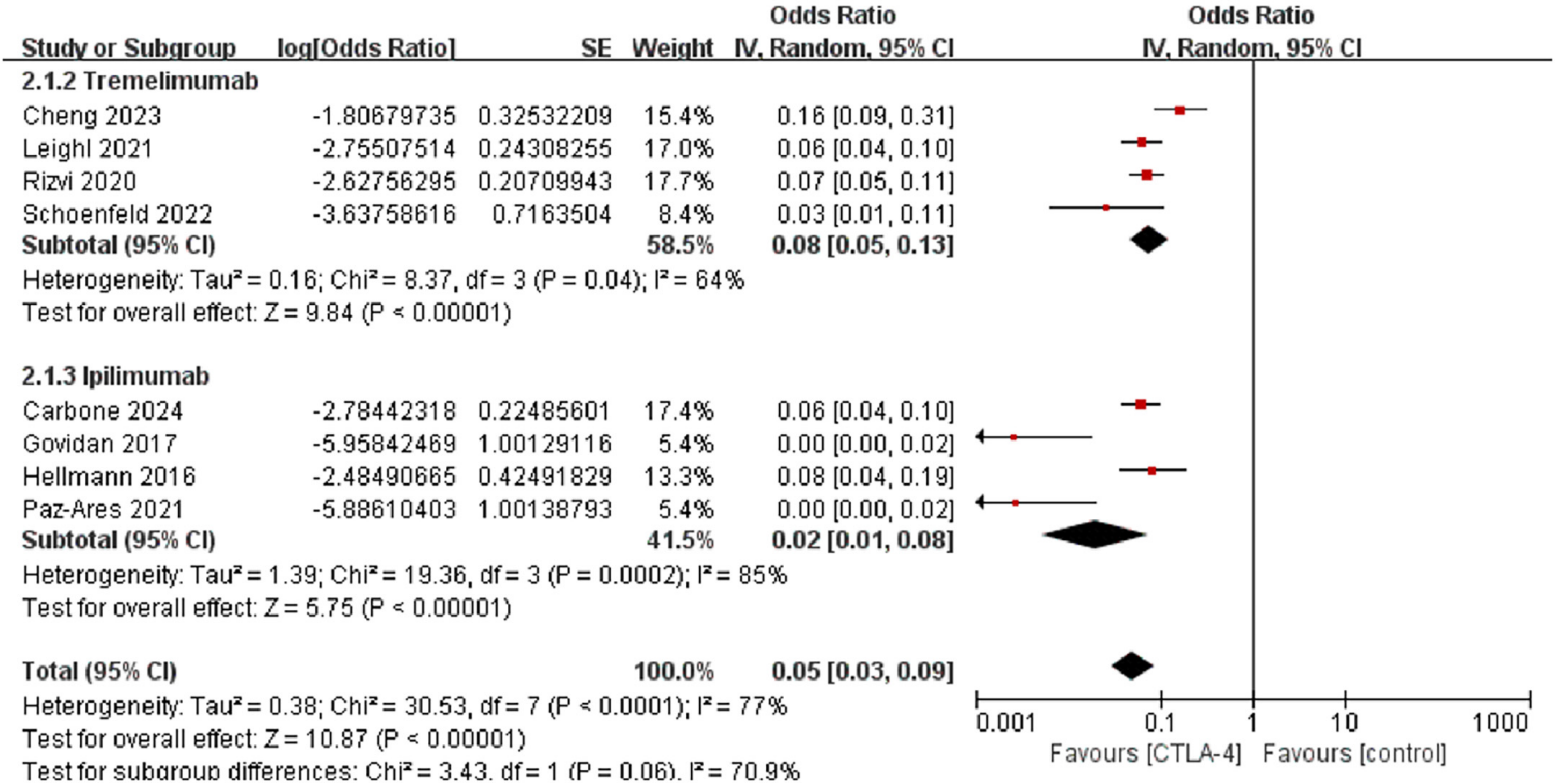
Forest plot comparing the incidence of any-grade pneumonitis between tremelimumab [8.0%, 95% CI (5.0%, 13.0%)] and ipilimumab [2.0%, 95% CI (1.0%, 8.0%)].

We further evaluated the risk of pneumonitis associated with different types of CTLA-4 inhibitors (tremelimumab and ipilimumab) in the treatment of NSCLC. The results showed that the incidence of all-grade pneumonitis among patients treated with tremelimumab was 8.0% [95% CI (5.0%, 13.0%)], with moderate heterogeneity (I^2^ = 64%, *p* < 0.05. In contrast, the incidence of all-grade pneumonitis among patients treated with ipilimumab was 2.0% [95% CI (1.0%, 8.0%)], with a similar level of moderate heterogeneity (I^2^ = 77%, *p* < 0.05), and a random-effects model was applied. These findings suggest that the incidence of all-grade pneumonitis is higher in patients treated with tremelimumab compared to those treated with ipilimumab (Figure 8).

**FIGURE 9:**
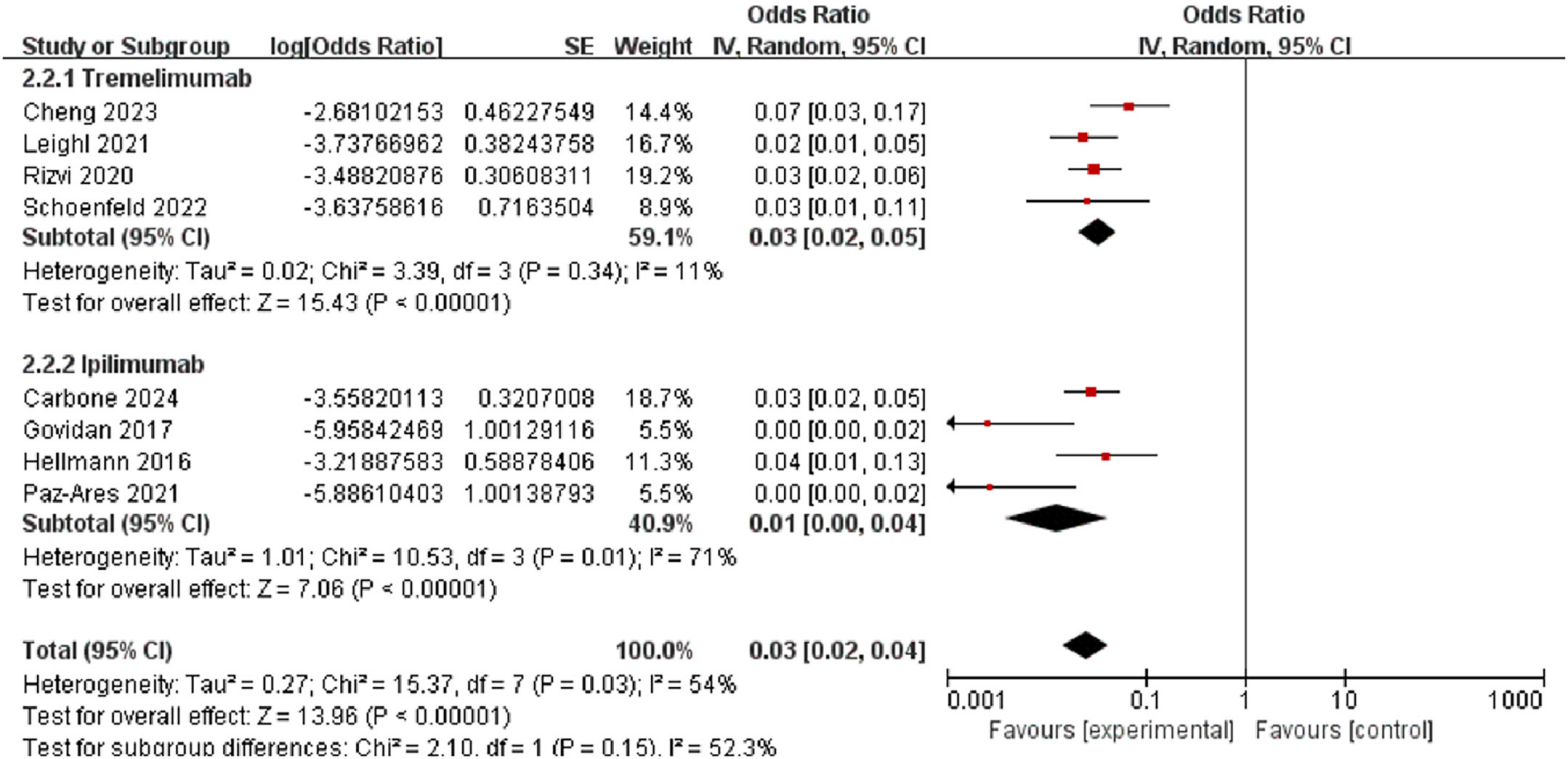
Forest plot comparing the incidence of high-grade (grade 3) pneumonitis between tremelimumab [3.0%, 95% CI (2.0%, 5.0%)] and ipilimumab [1.0%, 95% CI (0%, 4.0%)].

The incidence of high-grade pneumonitis among patients treated with tremelimumab was 3.0% [95% CI (2.0%, 5.0%)], with low heterogeneity (I^2^ = 11%, *P* = 0.34). In comparison, the incidence of high-grade pneumonitis among patients treated with ipilimumab was 1.0% [95% CI (0%, 4.0%)], with moderate heterogeneity (I^2^ = 71%, *P* = 0.001), and a random-effects model was applied. These results indicate that the incidence of high-grade pneumonitis is higher in patients treated with tremelimumab compared to those treated with ipilimumab (Figure 9).

## 4 Discussion

Immunotherapy with ICIs has revolutionized the treatment landscape for various malignancies, including NSCLC. These ICIs, which are monoclonal antibodies, modulate the immune system to enhance T-cell- mediated cytotoxicity, thereby promoting more effective anti-tumor immune responses (29, 30). However, overstimulation of the immune system due to these therapies can lead to a range of treatment-related adverse events, with pneumonitis being one of the most concerning due to its potential severity (31–33). A comparison of pneumonitis incidence across different classes of ICIs may further highlight the clinical relevance of focusing on CTLA-4 inhibitors. Previous studies have shown that PD-1/PD-L1 inhibitors are generally associated with a higher incidence of pneumonitis compared to CTLA-4 inhibitors (34). However, when these agents are used in combination, the risk of pneumonitis increases significantly.

### 4.1 CTLA-4 inhibitors and their mechanism of action

CTLA-4 is predominantly expressed by activated CD4+ and CD8+ T cells, and it is also found on regulatory T cells (Tregs) (35). Blocking CTLA-4 can enhance T cell activity, inhibit the regulatory function of Tregs, and thereby strengthen the immune response to tumors (36). Studies have shown that CTLA-4 inhibitors can prolong OS and PFS in patients with NSCLC, while also improving their overall response rates.

Unlike adverse events associated with traditional chemotherapy or targeted therapy, irAEs are organ- specific and often dose-independent (37). Pneumonitis is one of the most severe irAEs, and in some cases, it can be life-threatening (38). A thorough understanding of immune- associated pneumonitis and its clinical management is crucial for the safe and widespread use of immune checkpoint inhibitors.

### 4.2 Incidence of pneumonitis with CTLA-4 inhibitors

This meta-analysis, which included 4,164 patients treated with CTLA-4 inhibitors (either as monotherapy or in combination therapy), found an overall incidence of any-grade pneumonitis of 4.0% [95% CI (2.2%, 5.8%)]. This finding is consistent with previous clinical trial data, which indicate that the incidence of pulmonary irAEs in NSCLC patients ranges from 3% to 5% (37, 38). In contrast, a retrospective study of 205 NSCLC patients found that 19% experienced pneumonitis during PD-1/PD-L1 blockade (39), suggesting that CTLA-4 inhibitors may result in a lower incidence of pneumonitis compared to PD-1/PD-L1 inhibitors (40).

### 4.3 Comparison between CTLA-4 inhibitor types

Our subgroup analysis revealed notable differences between individual CTLA-4 inhibitors. Tremelimumab was associated with a higher incidence of both any-grade (8.0% vs. 2.0%) and high- grade (3.0% vs. 1.0%) pneumonitis compared to ipilimumab. This finding is clinically significant and may influence treatment decisions, particularly for patients with pre-existing pulmonary conditions or risk factors for developing pneumonitis.

Ipilimumab, the first monoclonal anti-CTLA-4 antibody, blocks the interaction between CTLA-4 and its ligands. In an open-label Phase III trial for patients with stage IV or recurrent NSCLC (NCT02477826), the combination of ipilimumab plus nivolumab showed a median survival of 17.1 months compared to 13.9 months in the chemotherapy group (23). These findings suggest that ipilimumab improves OS and PFS in NSCLC patients, particularly in combination therapy.

Tremelimumab, a fully humanized IgG2 monoclonal antibody, activates T cells by targeting CTLA-4 (41). The open-label Phase III POSEIDON study evaluated tremelimumab in combination with durvalumab and with chemotherapy alone in first-line metastatic NSCLC (mNSCLC). Results showed that tremelimumab combined with durvalumab and chemotherapy significantly improved OS and PFS compared to chemotherapy alone (10).

The markedly higher incidence of pneumonitis observed with tremelimumab compared to ipilimumab warrants careful attention when selecting treatment for NSCLC patients, particularly those with pre-existing pulmonary conditions. This difference may be due to structural and pharmacokinetic differences between the two agents. Tremelimumab is a fully humanized IgG2 monoclonal antibody, whereas ipilimumab is of the IgG1 subclass—this distinction may affect immune effector functions, tissue penetration, and Fc receptor binding, thereby influencing toxicity profiles (42, 43). Furthermore, tremelimumab is commonly administered alongside durvalumab using fixed-dose or induction-intensified regimens, such as those employed in the POSEIDON trial, which may contribute to elevated immune activation and pneumonitis risk (44). Differences in trial populations may also play a role; for instance, a greater proportion of patients with underlying lung disease or prior thoracic radiotherapy in tremelimumab-treated cohorts may predispose these individuals to pneumonitis (45). Further mechanistic studies are needed to better elucidate the biological pathways underlying tremelimumab-associated pneumonitis, particularly in relation to cytokine signaling and pulmonary immune microenvironment.

### 4.4 Comparison with control group

Our analysis comparing the incidence of pneumonitis between patients receiving CTLA-4 inhibitors and control groups further supports the association between CTLA-4 inhibitors and pneumonitis. Patients treated with CTLA-4 inhibitors had a significantly higher incidence of any-grade pneumonitis [OR = 3.00, 95% CI (1.60, 5.64); *p* < 0.01]. However, the difference in high-grade pneumonitis, while numerically higher, did not reach statistical significance [RR = 1.79, 95% CI (0.83, 3.85); *P* = 0.14].

All studies included in this analysis used CTLA-4 inhibitors in combination with other treatments such as chemotherapy or radiotherapy for NSCLC. The higher incidence of pneumonitis in the CTLA-4 inhibitor group may be partly attributable to the additive pulmonary toxicity from combined treatments. In a randomized Phase III clinical trial, NSCLC patients treated with durvalumab in combination with tremelimumab had a higher incidence of any-grade and high-grade pneumonitis compared to those treated with monotherapy (6.7% versus 2.2% for any-grade, and 2.2% versus 1.1% for high-grade) (10), which aligns with our findings.

### 4.5 Attribution of pulmonary toxicity and role of monotherapy

Although this meta-analysis evaluates pneumonitis in CTLA-4–based regimens, it remains challenging to attribute the observed pulmonary toxicity solely to CTLA-4 inhibition. Most clinical studies involve combination therapies with PD-1/PD-L1 inhibitors, making it difficult to disentangle the respective contributions of each agent. Available data from early trials of CTLA-4 monotherapy, such as ipilimumab in melanoma, suggest a relatively low incidence of pneumonitis (typically <1%) (46). For example, pivotal pre-marketing trials of ipilimumab monotherapy in melanoma, such as MDX010-20 and CA184-024, reported pneumonitis rates below 1%, supporting the hypothesis of lower pulmonary toxicity with monotherapy (27, 28). However, in NSCLC, evidence from monotherapy trials is sparse (47), limiting our ability to draw definitive conclusions. Retrospective analyses and meta-analyses have indicated that the risk of pneumonitis increases significantly when CTLA-4 inhibitors are combined with PD-1/PD-L1 blockade, suggesting a potential synergistic effect on immune-mediated lung toxicity (48). These findings highlight the importance of stratifying future analyses by treatment modality to better assess risk. Mechanistically, CTLA-4 and PD-1/PD-L1 differ in both timing and anatomical sites of immune modulation. CTLA-4 primarily acts in the early stages of T-cell activation within lymphoid organs, whereas PD-1/PD-L1 functions at later stages, mainly within the tumor microenvironment. Consequently, CTLA-4 blockade may lead to broader systemic T-cell activation (49), while PD-1/PD-L1 inhibition is more likely to unmask localized immune reactions within peripheral tissues, such as the lungs (50). These mechanistic distinctions may partly explain the higher pneumonitis incidence commonly observed with PD-1/PD-L1 inhibitors, especially in lung cancer patients. Therefore, while CTLA-4 may contribute to immune-related pneumonitis, current evidence suggests that the increased risk seen in combination regimens is more likely driven by the PD-1/PD-L1 component.

### 4.6 Limitations

This meta-analysis has several limitations. First, the number of included RCTs was small (*n* = 9), which limits statistical power. Second, strict inclusion criteria in most studies may reduce generalizability to real-world populations. Third, the lack of standardized diagnostic criteria for pneumonitis may have introduced inconsistency in outcome definitions. Additionally, treatment heterogeneity—monotherapy versus combination regimens—could influence pooled estimates.

Stratified analyses by region or patient characteristics (e.g., age, smoking history) were not feasible due to limited and inconsistently reported data (19, 20, 24). Subgrouping these few studies would yield imprecise estimates, as noted in the Cochrane Handbook (17). Key variables such as smoking status, lung comorbidities, or prior thoracic radiotherapy were often reported in aggregated formats, limiting assessment of known risk factors for pneumonitis (20, 22, 37, 51). Another important limitation is that our pooled analysis combined data from monotherapy and combination regimens, which may obscure differential risks associated with each approach. Additionally, randomized controlled trials often involve highly selected patient populations with fewer comorbidities and stricter eligibility criteria, potentially leading to an underestimation of pneumonitis incidence compared to real-world cohorts. This highlights the need for further real-world studies to validate and contextualize these findings.

## 5 Conclusion

This meta-analysis demonstrates that the use of CTLA-4 inhibitors in NSCLC treatment is associated with a notable incidence of pneumonitis (4.0% for any-grade, 1.6% for high-grade). Importantly, our subgroup analysis revealed that tremelimumab is associated with a higher incidence of both any-grade (8.0%) and high-grade (3.0%) pneumonitis compared to ipilimumab (2.0% and 1.0%, respectively).

These findings have important clinical implications, highlighting the need for vigilant monitoring for pneumonitis in NSCLC patients receiving CTLA-4 inhibitors, particularly tremelimumab. The significant difference between tremelimumab and ipilimumab in pneumonitis incidence may influence treatment selection, especially for patients with pre-existing pulmonary conditions or other risk factors for developing pneumonitis. Future research should focus on elucidating the underlying mechanisms of CTLA-4 inhibitor- induced pneumonitis, identifying predictive biomarkers for pneumonitis risk, and developing optimal prevention and management strategies. Additionally, real-world studies would provide valuable insights into the incidence and characteristics of pneumonitis outside the controlled setting of clinical trials.

## Data Availability

The original contributions presented in this study are included in this article/Supplementary material, further inquiries can be directed to the corresponding authors.
