# Supplementary material for: Incidence of pneumonitis with CTLA-4 inhibitors in non-small cell lung cancer: a systematic review and meta-analysis
Source: Front Med (Lausanne). 2025 Aug 6;12:1614442. doi: 10.3389/fmed.2025.1614442 (PMC12364688; doi:10.3389/fmed.2025.1614442)
Supplement: Supplementary file 1 [file Table_1.docx]

Table S1 Search terms used for retriving included studies

| Database | Search terms |
| --- | --- |
| Pubmed | (("Carcinoma, Non-Small-Cell Lung"[Mesh]) OR (((((((((((Carcinomas, Non-Small-Cell Lung) OR (Carcinomas, Non-Small-Cell Lung)) OR (Lung Carcinoma, Non-Small-Cell)) OR (Lung Carcinomas, Non-Small-Cell)) OR (Non-Small-Cell Lung Carcinomas)) OR (Carcinoma, Non-Small Cell Lung)) OR (Non-Small Cell Lung Cancer)) OR (Non-Small-Cell Lung Carcinoma)) OR (Non Small Cell Lung Carcinoma)) OR (Nonsmall Cell Lung Cancer)) OR (Non-Small Cell Lung Carcinoma))) AND ((((((((((((Anti-CTLA-4 MAb Ipilimumab) OR (Ipilimumab)) OR (Anti CTLA 4 MAb Ipilimumab)) OR (Ipilimumab, Anti-CTLA-4 MAb)) OR (MDX 010)) OR (MDX-010)) OR (MDX010)) OR (MDX-CTLA-4)) OR (MDX CTLA 4)) OR (Yervoy)) OR (("tremelimumab" [Supplementary Concept]) OR (((((((((ticilimumab) OR (Tremelimumab)) OR (CP-675,206)) OR (CP 675206)) OR (CP-675206)) OR (CP675206)) OR (CP 675)) OR (CP-675)) OR (CP675 cpd)))) OR (("CTLA-4 Antigen"[Mesh]) OR (((((((((((((CTLA-4 Antigen) OR (Antigen, CTLA-4)) OR (CTLA 4 Antigen)) OR (CTLA-4 Protein)) OR (CTLA 4 Protein)) OR (Cytotoxic T-Lymphocyte Antigen 4)) OR (Cytotoxic T Lymphocyte Antigen 4)) OR (Antigens, CD152)) OR (CD152 Antigens)) OR (CD152 Antigen)) OR (Antigen, CD152)) OR (Cytotoxic T-Lymphocyte-Associated Antigen 4)) OR (Cytotoxic T Lymphocyte Associated Antigen 4)))) |
| **EMBASE** | 'carcinoma, non-small-cell lung'/exp OR 'carcinoma, non-small-cell lung' OR (('carcinoma,'/exp OR carcinoma,) AND 'non small cell' AND ('lung'/exp OR lung)) OR (carcinomas, AND 'non small cell' AND lung) OR (lung AND carcinoma, AND 'non small cell') OR (lung AND carcinomas, AND 'non small cell') OR ('non small cell' AND lung AND carcinomas) OR (carcinoma, AND 'non small' AND cell AND lung) OR ('non small' AND cell AND lung AND cancer) OR ('non small cell' AND lung AND carcinoma) OR (non AND small AND cell AND lung AND carcinoma) OR (nonsmall AND cell AND lung AND cancer) OR ('non small' AND cell AND lung AND carcinoma)  AND  'tremelimumab'/exp OR tremelimumab OR 'cp 675,206' OR (cp AND 675206) OR 'cp 675206' OR cp675206 OR (cp AND 675) OR 'cp 675' OR (cp675 AND cpd) OR ticilimumab  OR  'ipilimumab'/exp OR ipilimumab OR ('anti ctla 4' AND mab AND ipilimumab) OR (anti AND ctla AND 4 AND mab AND ipilimumab) OR (ipilimumab, AND 'anti ctla 4' AND mab) OR (mdx AND 010) OR 'mdx 010' OR mdx010 OR 'mdx ctla 4' OR (mdx AND ctla AND 4) OR yervoy  OR  'ctla-4 antigen'/exp OR 'ctla-4 antigen' OR (('ctla 4'/exp OR 'ctla 4') AND ('antigen'/exp OR antigen)) OR (antigen, AND 'ctla 4') OR (ctla AND 4 AND antigen) OR ('ctla 4' AND protein) OR (ctla AND 4 AND protein) OR (cytotoxic AND 't lymphocyte' AND antigen AND 4) OR (cytotoxic AND t AND lymphocyte AND antigen AND 4) OR (antigens, AND cd152) OR (mdx AND ctla AND 4) OR (cd152 AND antigens) OR (cd152 AND antigen) OR (antigen, AND cd152) OR (cytotoxic AND 't lymphocyte associated' AND antigen AND 4) OR (cytotoxic AND t AND lymphocyte AND associated AND antigen AND 4) |
| **Cochrane** | ID Search Hits  #1 MeSH descriptor: [Carcinoma, Non-Small-Cell Lung] explode all trees 6655  #2 (Carcinoma, Non-Small-Cell Lung or Carcinomas, Non-Small-Cell Lung or Lung Carcinoma, Non-Small-Cell or Lung Carcinomas, Non-Small-Cell or Non-Small-Cell Lung Carcinomas or Carcinoma, Non-Small Cell Lung or Non-Small Cell Lung Cancer or Non-Small-Cell Lung Carcinoma or Non Small Cell Lung Carcinoma or Nonsmall Cell Lung Cancer or Non-Small Cell Lung Carcinoma):ti,ab,kw (Word variations have been searched) 17054  #3 #1 or #2 17054  #4 (Tremelimumab or CP-675,206 or CP 675206 or CP-675206 or CP675206 or CP 675 or CP-675 or CP675 cpd or Ticilimumab):ti,ab,kw (Word variations have been searched) 492  #5 MeSH descriptor: [Ipilimumab] explode all trees 569  #6 (Ipilimumab or Anti-CTLA-4 MAb Ipilimumab or Anti CTLA 4 MAb Ipilimumab or Ipilimumab, Anti-CTLA-4 MAb or MDX 010 or MDX-010 or MDX010 or MDX-CTLA-4 or MDX CTLA 4 or Yervoy):ti,ab,kw (Word variations have been searched) 1960  #7 #5 or #6 1960  #8 MeSH descriptor: [CTLA-4 Antigen] explode all trees 88  #9 (CTLA-4 Antigen or Antigen, CTLA-4 or CTLA 4 Antigen or CTLA-4 Protein or CTLA 4 Protein or Cytotoxic T-Lymphocyte Antigen 4 or Cytotoxic T Lymphocyte Antigen 4 or Antigens, CD152 or CD152 Antigens or CD152 Antigen or Antigen, CD152 or Cytotoxic T-Lymphocyte-Associated Antigen 4 or Cytotoxic T Lymphocyte Associated Antigen 4):ti,ab,kw (Word variations have been searched) 645  #10 #8 or #9 645  #11 #4 or #7 or #10 2801  #12 #3 and #11 441 |

Table S2 Selected clinical trials for meta-analysis

| General information of the study | | | | Characteristics of the studies | | | | Outcome effect data | | | Original research methodology | | |
| --- | --- | --- | --- | --- | --- | --- | --- | --- | --- | --- | --- | --- | --- |
| Title | First Author | Year | Groups | N Patients | All N Patients | age | Disease classification | Number of pneumonia cases | Pneumonia grade 1-5 (number of people) | Pneumonia grade 3-5(number of people) | Follow-up time(years) | Type of study | Phase of study |
| A phase Ib study evaluating the safety and efficacy of IBI310 plus sintilimab in patients with advanced non-­small-­cell lung cancer who have progressed after anti-­PD-­1/L1 therapy | Yuguang Zhao | 2024 | IBI310 1 mg/kg plus sintilimab | 15 | 30 | 66.0(57-74) | Adenocarcinoma (8 cases, 53.3%), squamous cell carcinoma (6 cases, 40.0%) | 2 | 2 | 0 | 4.2 | RCT | Ib |
|  |  |  | IBI310 3 mg/kg plus sintilimab | 15 |  | 58.0(42-72) | Squamous cell carcinoma (12 cases, 80.0%), adenocarcinoma (3 cases, 20%) | 3 | 3 | 0 | 5.6 |  |  |
| Durvalumab plus tremelimumab alone or in combination with low-dose or hypofractionated radiotherapy in metastatic non-small-cell lung cancer refractory to previous PD(L)-1 therapy: an open-label, multicentre, randomised,phase 2 trial | Jonathan D Schoenfeld | 2022 | Durvalumab–tremelimumab plus hypofractionated radiotherapy (n=26) | 26 | 78 | 65.0 (60–70) | Adenocarcinoma/Squamous/Not specified 19 (73%)/21 (81%)/16 (62%) | 1 | 1 | 1 | - | RCT | II |
|  |  |  | Durvalumab–tremelimumab plus low-dose radiotherapy (n=26) | 26 |  | 65.0 (60–73) | 3 (12%)/1 (4%)/5 (19%) | 0 | 0 | 0 | - |  |  |
|  |  |  | Durvalumab–tremelimumab alone (n=26) | 26 |  | 65.0 (57–72) | 4 (15%)/4 (15%)/5 (19%) | 1 | 1 | 1 | - |  |  |
| Durvalumab With or Without Tremelimumab vs Standard Chemotherapy in First-line Treatment ofMetastatic Non–Small Cell Lung Cancer The MYSTIC Phase 3 Randomized Clinical Trial | Naiyer A. Rizvi | 2020 | Durvalumab Monotherapy (n = 369) | 163 | 488 | 64.0 (32-84) | Squamous52 (31.9)/53 (32.5)/52 (32.1)；Nonsquamous111 (68.1)/110 (67.5)/110 (67.9) | 8 | 8 | 5 | - | RCT | III |
|  |  |  | Durvalumab + Tremelimumab (n = 371) | 163 |  | 65.0 (34-87) |  | 25 | 25 | 11 | - |  |  |
|  |  |  | Chemotherapy(n = 352) | 162 |  | 64.5 (35-85) |  | 5 | 5 | 2 | - |  |  |

Table S2 continued

| General information of the study | | | | Characteristics of the studies | | | | Outcome effect data | | | Original research methodology | | |
| --- | --- | --- | --- | --- | --- | --- | --- | --- | --- | --- | --- | --- | --- |
| Title | First Author | Year | Groups | N Patients | All N Patients | age | Disease classification | Number of pneumonia cases | Pneumonia grade 1-5 (number of people) | Pneumonia grade 3-5(number of people) | Follow-up time(years) | Type of study | Phase of study |
| First-line nivolumab plus ipilimumab combined with two cycles of chemotherapy in patients with non-small-cell lung cancer (CheckMate 9LA): an international, randomised,open-label, phase 3 trial | Luis Paz-Ares | 2021 | Nivolumab plus ipilimumab with two cycles of chemotherapy group (n=361); | 361 | 719 | 65.0 (59.0–70.0) | Squamous113 (31%)/111 (31%)；Non-squamous 248 (69%)/247 (69%) | 1 | 1 | 1 | - | RCT | III |
|  |  |  | Chemotherapy group (n=358) | 358 |  | 65.0 (58.0–70.0) |  | 0 | 0 | 0 | - |  |  |
| Nivolumab plus ipilimumab as fi rst-line treatment for advanced non-small-cell lung cancer (CheckMate 012): results of an open-label, phase 1, multicohort study | Matthew D Hellmann | 2016 | nivolumab 3 mg/kg every 2 weeks plus ipilimumab 1 mg/kg every 12 weeks（n=38）; | 38 | 78 | 68.0 (58−73) | Non-squamous 31 (82%)/33 (85%)；Squamous 7 (18%)/6 (15%) | 4 | 4 | 2 | - | RCT | I |
|  |  |  | nivolumab 3 mg/kg every 2 weeks plus ipilimumab 1 mg/kg every 6 weeks(n=40) | 40 |  | 62.0 (57−73) |  | 2 | 2 | 1 | - |  |  |
| NEPTUNE China cohort: First-line durvalumab plus tremelimumab in Chinese patients with metastatic non-small-cell lung cancer | Ying Cheng a | 2023 | Durvalumab + tremelimumab (n =77)； | 78 | 160 | 61.0 (35–72) | Squamous 24 (30.8)/25 (30.5)；Non-squamous 54 (69.2)/57 (69.5) | 11 | 11 | 5 | - | RCT | III |
|  |  |  | Chemotherapy (n = 78)） | 82 |  | 62.5 (35–77) |  | 3 | 3 | 3 | - |  |  |

Table S2 continued

| General information of the study | | | | Characteristics of the studies | | | | Outcome effect data | | | Original research methodology | | |
| --- | --- | --- | --- | --- | --- | --- | --- | --- | --- | --- | --- | --- | --- |
| Title | First Author | Year | Groups | N Patients | All N Patients | age | Disease classification | Number of pneumonia cases | Pneumonia grade 1-5 (number of people) | Pneumonia grade 3-5(number of people) | Follow-up time(years) | Type of study | Phase of study |
| Four-­year clinical update and treatment switching- adjusted outcomes with first-line nivolumab plus ipilimumab with chemotherapy for metastatic non-­small cell lung cancer in the CheckMate 9LA randomized trial | David P. Carbone | 2024 | Nivolumab plus ipilimumab with chemotherapy (n=361) | 361 | 719 | 65.0 (59.0–70.0) | Squamous113 (31%)；Non-squamous 248 (69%) | 21 | 21 | 10 | - | RCT | - |
|  |  |  | Chemotherapy (n=358) | 358 |  | - |  | - | - | - | - |  |  |
| Phase III Trial of Ipilimumab Combined With Paclitaxel and Carboplatin in Advanced Squamous Non–Small-Cell Lung Cancer | Ramaswamy Govindan | 2017 | Chemotherapy Plus Ipilimumab | 388 | 749 | 64 (28-84) |  | 1 | 1 | 1 | 12.5 | RCT | III |
|  |  |  | Chemotherapy Plus Placebo | 361 |  | 64 (28-85) |  | 2 | 2 | 2 | 11.8 |  |  |
| CCTG BR34: A Randomized Phase 2 Trial of Durvalumab and Tremelimumab With or Without Platinum-Based Chemotherapy in Patients With Metastatic NSCLC | Natasha B. Leighl | 2021 | Durvalumab +Tremelimumab + Chemotherapy (n =151) | 151 | 301 | 65.0 (27–79) |  | 9 | 9 | 3 | - | RCT | II |
|  |  |  | Durvalumab + Tremelimumab (n = 150) | 150 |  | 63.0 (38–87) |  | 9 | 9 | 4 | - |  |  |

...........


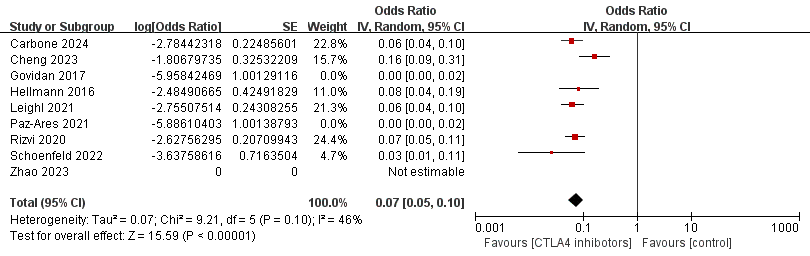


**Supplementary Figure 1.** Forest plot of pneumonitis incidence in CTLA-4 inhibitor studies after excluding Govindan2017 and Paz-Ares2021 (I² = 46%, p = 0.10).

..


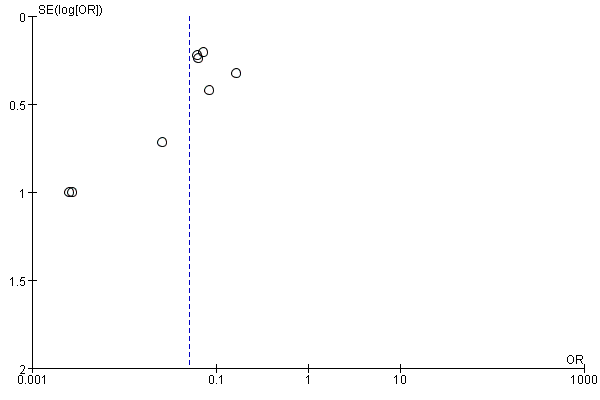


**Supplementary Figure 2. Funnel Plot of Pneumonitis Incidence in CTLA-4 Inhibitor Studies for Publication Bias Assessment**

This funnel plot displays the standard error of the log odds ratio (SE[log(OR)]) on the y-axis against the odds ratio (OR) on the x-axis for the nine studies included in the meta-analysis. The vertical dashed line represents the pooled odds ratio. The plot is used to visually assess potential publication bias, with asymmetry indicating possible bias**.**
